# Diagnostic yield and accuracy of coronary CT angiography after abnormal nuclear myocardial perfusion imaging

**DOI:** 10.1038/s41598-018-27347-8

**Published:** 2018-06-15

**Authors:** Felix G. Meinel, U. Joseph Schoepf, Jacob C. Townsend, Brian A. Flowers, Lucas L. Geyer, Ullrich Ebersberger, Aleksander W. Krazinski, Wolfgang G. Kunz, Kolja M. Thierfelder, Deborah W. Baker, Ashan M. Khan, Valerian L. Fernandes, Terrence X. O’Brien

**Affiliations:** 1 Heart and Vascular Center, Medical University of South Carolina, Charleston, SC USA; 2 Department of Diagnostic and Interventional Radiology, Rostock University Medical Center, Rostock, Germany; 3 Division of Cardiology, Department of Medicine, Medical University of South Carolina, Charleston, SC USA; 4 Birmingham Heart Clinic, Birmingham, AL US; 5 Center for Radiology and Neuroradiology, Klinikum Ingolstadt, Ingolstadt, Germany; 6 Department of Radiology, Ludwig-Maximilians-University Hospital, Munich, Germany; 7 Charles George Veterans Affairs Medical Center, Asheville, NC USA; 8 Department of Radiology, Ralph H. Johnson Veterans Affairs Medical Center, Charleston, SC USA; 9 Department of Medicine, Ralph H. Johnson Veterans Affairs Medical Center, Charleston, SC USA

## Abstract

We aimed to determine the diagnostic yield and accuracy of coronary CT angiography (CCTA) in patients referred for invasive coronary angiography (ICA) based on clinical concern for coronary artery disease (CAD) and an abnormal nuclear stress myocardial perfusion imaging (MPI) study. We enrolled 100 patients (84 male, mean age 59.6 ± 8.9 years) with an abnormal MPI study and subsequent referral for ICA. Each patient underwent CCTA prior to ICA. We analyzed the prevalence of potentially obstructive CAD (≥50% stenosis) on CCTA and calculated the diagnostic accuracy of ≥50% stenosis on CCTA for the detection of clinically significant CAD on ICA (defined as any ≥70% stenosis or ≥50% left main stenosis). On CCTA, 54 patients had at least one ≥50% stenosis. With ICA, 45 patients demonstrated clinically significant CAD. A positive CCTA had 100% sensitivity and 84% specificity with a 100% negative predictive value and 83% positive predictive value for clinically significant CAD on a per patient basis in MPI positive symptomatic patients. In conclusion, almost half (48%) of patients with suspected CAD and an abnormal MPI study demonstrate no obstructive CAD on CCTA.

## Introduction

Patients with clinical symptoms concerning for angina commonly undergo myocardial perfusion imaging (MPI). Abnormal results deemed significant are typically referred for invasive coronary angiography (ICA). One concern of this approach is that the overall diagnostic yield of finding obstructive coronary artery disease (CAD) in patients undergoing elective ICA in patients can be relatively low, with a range of diagnostic yields which can be as high as 60% of patients demonstrating no obstructive CAD^1^. This reflects in part the limited correlation between anatomical and functional assessments of CAD as well as other factors such as diagnostic accuracy. It is increasingly being recognized that there may not be a single best testing strategy and that different patient groups should be studied to determine the optimal diagnostic approach.

Cardiac catheterization carries a relatively small but not negligible risk of major complications^2^. There has been much effort put forward trying to identify patients who would be most likely to benefit from ICA and to accurately determine which patients do not require an invasive procedure. MPI is an established approach but questions remain whether accuracy can be improved and whether unique patient subsets should be evaluated differently. Therefore, we tested the hypothesis of whether using coronary CT angiography (CCTA) as a gatekeeper to ICA in the setting of an abnormal MPI could reduce the number of ICAs by reliably identifying false positive studies and improve patient safety while maintaining diagnostic accuracy. While recent appropriateness criteria consider CCTA an acceptable alternative to ICA in patients with diagnostically ambiguous MPI studies^3^, the data available on the diagnostic yield and the accuracy of CCTA in this patient population is largely limited to retrospective analyses and specific patient groups. We sought to expand these results by studying the diagnostic accuracy of CCTA in a unique population of patients at higher risk for having obstructive CAD. Part of this rationale is that there is limited data available in patients presenting with both symptoms suggestive of CAD and who have also subsequently had an abnormal MPI leading to a referral for ICA. Therefore, we studied the diagnostic yield and accuracy of CCTA in patients already referred for ICA based on clinical indications suggesting obstructive CAD after an abnormal MPI stress test.

## Methods

### Ethics and informed consent

This study was approved by the institutional review boards of the Medical University of South Carolina and the Ralph H. Johnson Veterans Affairs Medical Center both in Charleston, South Carolina. All patients provided written informed consent prior to enrollment. The study was conducted in compliance with the Declaration of Helsinki as well as HIPAA and institutional regulations.

### Study design

The study was designed as a prospective, single-arm diagnostic study recruiting patients from two affiliated, neighboring centers, the Medical University of South Carolina and the Ralph H. Johnson Veterans Affairs Medical Center. The target recruitment population included patients with a clinical indication for ICA due to an abnormal recent nuclear stress MPI study performed for suspected CAD. Eligible patients were recruited and screened only after being referred and scheduled for ICA. Patients underwent an investigational CCTA before their ICA. Inclusion criteria consisted of (1) adults aged 30–90 who, (2) were supected of having CAD, (3) were independently previously scheduled to undergo a clinically indicated ICA after an abnormal MPI and, (4) consented to undergo an investigational CCTA prior to their ICA. Exclusion criteria consisted of (1) unstable symptoms, vital signs or electrocardiogram; (2) prior coronary artery bypass surgery; (3) creatinine >2.0 mg/dl; (4) potential pregnancy; or (5) prior reaction to iodinated contrast material. For ethical reasons, patients were excluded in whom the acquisition of a CCTA prior to their ICA would have delayed the ICA procedure by more than 6 hours due to scheduling difficulties. To minimize the risk of selection bias, all patients clinically referred for elective ICA specifically based on an abnormal MPI were screened for eligibility. Recruitment was limited to the 2 days weekly when research CCTA resources were available. Recruitment and study procedures were kept uniform throughout the enrollment period. One hundred patients were prospectively enrolled between September 2008 and September 2013 with a 20-month hiatus because of a lack of study coordinator resources. CCTA examinations were performed no more than 24 hours prior to ICA and were commonly done on the morning of the ICA. The median time interval between the abnormal nuclear stress MPI study and the CCTA and ICA was 22 days (interquartile range 13–37 days).

At the time of enrollment, demographic information, clinical and laboratory data were collected. Any regular medications taken for cardiovascular indications were also recorded. Blood samples were drawn and serum levels of creatinine, high density lipoprotein and low density lipoprotein measured.

### Nuclear stress myocardial perfusion imaging

Nuclear stress MPI was performed using standard stress/rest single photon emission computed tomography protocols with attenuation correction. Of the 100 nuclear stress imaging studies, 61 were performed using the Bruce or modified Bruce treadmill exercise protocol, with the remaining 39 examinations utilizing standard pharmacological stress protocols (adenosine, n = 24, regadenoson, n = 13, dipyridamole, n = 1, dobutamine, n = 1). A Symbia S (Siemens Healthcare, Hoffman-Estates, IL) dual head gamma camera was used for almost all acquisitions.

Analysis of MPI studies was performed independently twice; once before referral for ICA (and hence before screening) and again after enrollment in a blinded fashion by a nuclear medicine physician (AMK) or nuclear cardiologist (JCT, VLF, TXO). Perfusion abnormalities were recorded for 6 distinct myocardial regions of the left ventricle: anterior, lateral, posterior, inferior, septal and apical. For each region, perfusion abnormalities were specified as reversible, fixed or mixed. Additionally, the presence of transient ischemic dilatation (TID) or other significant abnormalities was recorded when present. Any differences between the two interpretations were adjudicated by a third nuclear cardiologist to achieve a final reading which was used for the data analysis (this was rarely required). Attenuation correction and summed stress scores were not routinely performed because of lack of availability.

### Coronary CT angiography

CCTA examinations were performed with use of 64-slice (SOMATOM Sensation 64, Siemens Healthcare, Forchheim, Germany) or dual-source CT system (SOMATOM Definition or Definition Flash, Siemens Healthcare). CCTA examinations were initially performed with retrospective ECG-gating. During the course of the study, prospective ECG-triggering was implemented for patients with regular heart rates. Tube voltage was 120 kV. Contrast enhancement was achieved with 60–120 ml of Omnipaque 350 mg/mL (GE Healthcare, Little Chalfont, UK), depending on the scan duration, followed by a saline chaser. Flow rate was 4–6 mL/s. The scan was started by bolus triggering in the ascending aorta. Patients were given 0.4–0.8 mg of nitroglycerin sublingually. Patients with a heart rate >65 beats per minute with no contraindications received up to 100 mg metoprolol orally 30–90 minutes prior to the examination or up to 20 mg of intravenous metoprolol tartrate immediately prior to the examination. The CCTA exam and ICA were separated by at least 6 hours in terms of contrast administration. All patients had normal renal function prior to imaging and none developed acute kidney injury after ICA.

All CCTA data were reconstructed with a section thickness of 0.75 mm and 0.5 mm increment using a vascular reconstruction kernel. Images were transferred to a dedicated post-processing workstation (Aquarius iNtuition, TeraRecon, San Mateo, CA) which allows for 3D multiplanar reformats, maximum intensity projections, as well as curved multiplanar reconstructions along vessel centerlines.

CT angiograms were evaluated in consensus by 2 experienced radiologists (FGM, LLG) who were blinded to the patients’ clinical data and the results of the nuclear myocardial perfusion imaging and catheterization. The degree of coronary artery stenosis was assessed individually for each vessel (left anterior descending coronary artery; left circumflex artery; right coronary artery) as harboring either no luminal narrowing (0% stenosis), non-obstructive coronary artery disease (1–49% stenosis), or potentially obstructive coronary artery disease (≥50% stenosis or occlusion). All branches with an assessable caliber were included with the respective vessel. The left main coronary artery and the ramus intermedius branch (if present) were counted towards the left anterior descending artery. If the lumen of a given vessel was not evaluable due to heavy calcifications or motion artifacts it was counted as positive for ≥50% stenosis according to an intention-to-diagnose approach in order to avoid the risk of overlooking any potentially significant stenoses.

### Invasive coronary angiography

Conventional x-ray coronary angiography was performed by standard techniques and in multiple projections. Angiograms were evaluated by 2 interventional cardiologists blinded to the CCTA data (VLF, JCT, BAF). Each major epicoronary vessel was assessed with 2-D planimetry confirming the estimation of the vessel to determine the degree of coronary artery stenosis which were categorized as harboring either no luminal narrowing (0% stenosis), non-obstructive coronary artery disease (1–49% stenosis), coronary artery disease with an intermediate degree of stenosis (50–69% stenosis) or severe stenosis (≥70% stenosis or occlusion). We defined clinically significant disease potentially warranting intervention as stenosis of ≥50% diameter narrowing in the left main coronary artery or ≥70% diameter narrowing in any other major epicoronary segment.

### Statistical analysis

All statistical analyses were performed using MedCalc Statistical Software version 12.7.2 (MedCalc Software bvba, Ostend, Belgium). A two-sided *P* < 0.05 was considered to indicate statistical significance. Continuous data was tested for normal distribution using the Kolmogorov-Smirnov test. Continuous data were displayed as mean ± standard deviation for normally distributed variables and as median and interquartile range for non-normally distributed data. Categorical data were displayed as absolute frequencies and proportions. To identify predictors of a positive CCTA (≥50% stenosis) following an abnormal MPI nuclear study, the following pre-specified parameters were compared between patients with and without ≥50% stenosis at CCTA: age, gender, number of cardiovascular risk factors (including hypertension, hyperlipidemia, diabetes mellitus, current or former smoking, family history of CAD, and obesity defined as BMI ≥ 30 kg/m^2^), regular beta-blocker use, stress method (treadmill vs. pharmacologic), type of perfusion abnormalities (reversible vs. mixed/fixed) and extensive perfusion abnormalities. Extensive perfusion abnormalities were predefined as involving ≥2 myocardial regions of the left ventricle (anterior, lateral, posterior, inferior, septal or apical). For intergroup comparison, the Chi squared test was used for categorical data and the t-test or Mann-Whitney test used for continuous data as appropriate. On a per patient level, we calculated the test characteristics (sensitivity, specificity, positive predictive value and negative predictive value) of a ≥50% stenosis on CT for the detection of clinically significant coronary artery disease, which we defined as any ≥70% stenosis or ≥50% left main stenosis on catheter angiograms. As a secondary analysis, the test characteristics for the detection of any ≥50% stenosis on catheter angiograms were also calculated.

### Data availability

The datasets generated and/or analyzed during the current study are available from the corresponding author on reasonable request.

## Results

The patient population consisted of 100 predominantly male patients (84%) of which 29% were African-American. There was a high prevalence of cardiovascular risk factors (Table 1). Indications for nuclear stress testing most commonly included chest pain (73%) and/or exertional dyspnea (29%). Seven patients were asymptomatic and underwent the nuclear stress test as part of a pre-operative evaluation; one was for a recent abnormal electrocardiogram, one for syncope and two for multiple cardiovascular risk factors. Medical therapy was typical for such a population being evaluated for high cardiovascular risk (Table 1).

**Table 1:** Patient Demographics (n = 100).

Age (years), mean ± standard deviation	59.6 ± 8.9
Males	84
Ethnicity	
Caucasian	69
African American	29
Asian	2
Risk Factors and Comorbidities	
Hypertension	82
Hyperlipidemia	82
Diabetes Mellitus	28
Current or Former Smoker	42
Family History of CAD	42
Prior myocardial infarction	3
Prior percutaneous coronary intervention	3
Prior stroke or transient ischemic attack	6
Obese (body mass index ≥ 30 kg/m^2^)	60
Laboratory parameters	
Creatinine, median (interquartile range) in mg/dL	1.0 (0.8–1.1)
HDL, median (interquartile range) in mg/dL	40 (33–47)
LDL, median (interquartile range) in mg/dL	100 (81–133)
Medications	
Aspirin	68
Statin	69
Other lipid lowering medication	17
Beta-blocker	46
ACE-inhibitor/ARB	49
Clopidogrel	14
Nitrate	13
Diuretic	32
Calcium channel blocker	34
Insulin	4
Oral anti-diabetic	20
Indications for nuclear stress test	
Chest pain/angina	73
Dyspnea	29
Preoperative evaluation	4
Other	4

ACE: angiotensin-converting enzyme; ARB: angiotensin receptor blocker; CAD: coronary artery disease; HDL: high density lipoprotein; LDL: low density lipoprotein.

Ninety-six of 100 patients demonstrated various perfusion abnormalities at nuclear stress testing. Of these, 82 were reversible compatible with ischemia, 2 were fixed compatible with infarction and 12 were partly reversible (mixed) (Table 2). Transient ischemic dilatation was noted in 18 patients; however, 16 of these also had significant reversible perfusion defects. Two patients did not have perfusion abnormalities or transient ischemic dilatation but their stress test was considered sufficiently abnormal to refer for ICA due to markedly reduced exercise capacity (n = 1) or chest pain with exercise (n = 1). Since all patients were referred for ICA on the basis of their MPI results prior to screening, all were included as an intention to treat analysis.

**Table 2:** Spectrum of findings at stress myocardial perfusion imaging (n = 100).

Perfusion abnormalities	96
Reversible perfusion defect (ischemia)	82
Fixed perfusion defect (infarction)	2
Mixed (partly reversible) perfusion defect	12
Transient ischemic dilatation	18
Reduced (<50%) ejection fraction	26
Other	2

CCTA results included fifty-four patients with potentially obstructive CAD (at least one ≥50% stenosis). Potentially obstructive lesions in a multi-vessel distribution (≥2 vessels) were present in 31 patients. Of the remaining 46 patients, 14 demonstrated mild (1–49%) luminal narrowing and 32 patients demonstrated no evidence of luminal narrowing (Table 3). Patients with a positive CCTA (≥50% stenosis) were significantly older on average than patients in whom CCTA demonstrated no potentially obstructive lesion (61.9 ± 8.8 vs. 57.0 ± 8.3 years, p = 0.0052) (Table 4).

**Table 3:** Spectrum of findings at CCTA (n = 100).

Potentially obstructive CAD (≥50% stenosis)	54
Single-vessel disease	23
Two-vessel disease	14
Three-vessel disease	17
Location of potentially obstructive CAD (≥50% stenosis)	
LAD (including LM)	42
LCX	31
RCA	29
Non-obstructive disease (1–49% stenosis)	14
No luminal narrowing (0% stenosis)	32

CAD: coronary artery disease; CCTA: coronary computed tomography angiography; LAD: left anterior descending coronary artery; LCX: left circumflex coronary artery; LM: left main coronary artery; RCA: right coronary artery.

**Table 4:** Predictors of a positive CCTA (≥50% stenosis) after an abnormal nuclear study.

Parameter	Negative CCTA (<50% stenosis) n = 46	Positive CCTA (≥50% stenosis) n = 54	p-value
Age	57.0 ± 8.3	61.9 ± 8.8	0.0052
Male gender	36 (78%)	48 (89%)	0.2415
Number of cardiovascular risk factors, median (interquartile range)	3 (3–4)	4 (3–4)	0.1116
Ergometric stress (vs. pharmacological stress)	29 (63%)	32 (59%)	0.8564
Reversible perfusion defect (vs. mixed or fixed defect)	41 (89%)	41 (76%)	0.1465
Extensive perfusion abnormalities	23 (50%)	36 (67%)	0.1376
Transient ischemic dilatation	7 (15%)	11 (20%)	0.6837

Patient characteristics, method of stress for nuclear perfusion imaging and findings at nuclear perfusion imaging are shown for the subgroups of patients without and with ≥50% stenosis on CCTA. Extensive perfusion abnormalities were predefined as abnormalities involving ≥2 myocardial regions (anterior, lateral, posterior, inferior, septal, apical). Age was compared using t-test for independent samples. The number of cardiovascular risk factors was compared using the non-parametric Mann-Whitney test. The frequency of categorical variables was compared using Chi squared test. CCTA: coronary computed tomography angiography.

On ICA, 45 patients demonstrated clinically significant CAD defined as any ≥70% stenosis or ≥50% left main stenosis. CCTA had 100% sensitivity and 84% specificity for the detection of clinically significant CAD, which translated into a 100% negative and 83% positive predictive value (Table 5a). As an additional analysis, the sensitivity of CCTA for the detection of any ≥50% stenosis on ICA was 96% with a specificity of 90% (Table 5b).

**Table 5:** Test characteristics of ≥50% stenosis on CCTA for the detection of (a) any ≥70% epicoronary stenosis or ≥50% left main stenosis on ICA and (b) any ≥50% epicoronary stenosis on ICA.

	(a)	(b)
Sensitivity (%)	100.0	96.1
	(92.1–100.0)	(86.5–99.5)
Specificity (%)	83.6	89.8
	(71.2–92.2)	(77.8–96.6)
Negative predictive value (%)	100.0	95.7
	(92.3–100.0)	(85.2–99.5)
Positive predictive value (%)	83.3	90.7
	(70.7–92.1)	(79.7–96.9)
True positives (n)	45	49
True negatives (n)	46	44
False positives (n)	9	5
False negatives (n)	0	2

CCTA: coronary computed tomography angiography; ICA: invasive coronary angiography. 95% confidence intervals are shown in parentheses.

**Figure 1:**
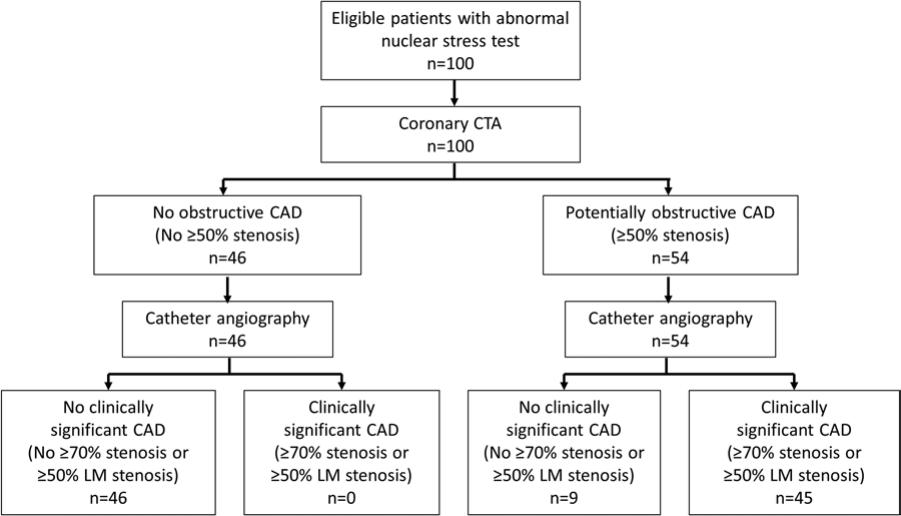
Flowchart demonstrating the results of CCTA and ICA. The presence of ≥50% stenosis was used to define a positive CCTA study as commonly used in clinical routine. We assessed the diagnostic accuracy of CCTA for the detection of clinically significant coronary artery disease, which we defined as any ≥70% stenosis or ≥50% left main stenosis on catheter angiograms. CAD = coronary artery disease, CCTA = coronary computed tomography angiography, LM = left main coronary artery.

**Figure 2:**
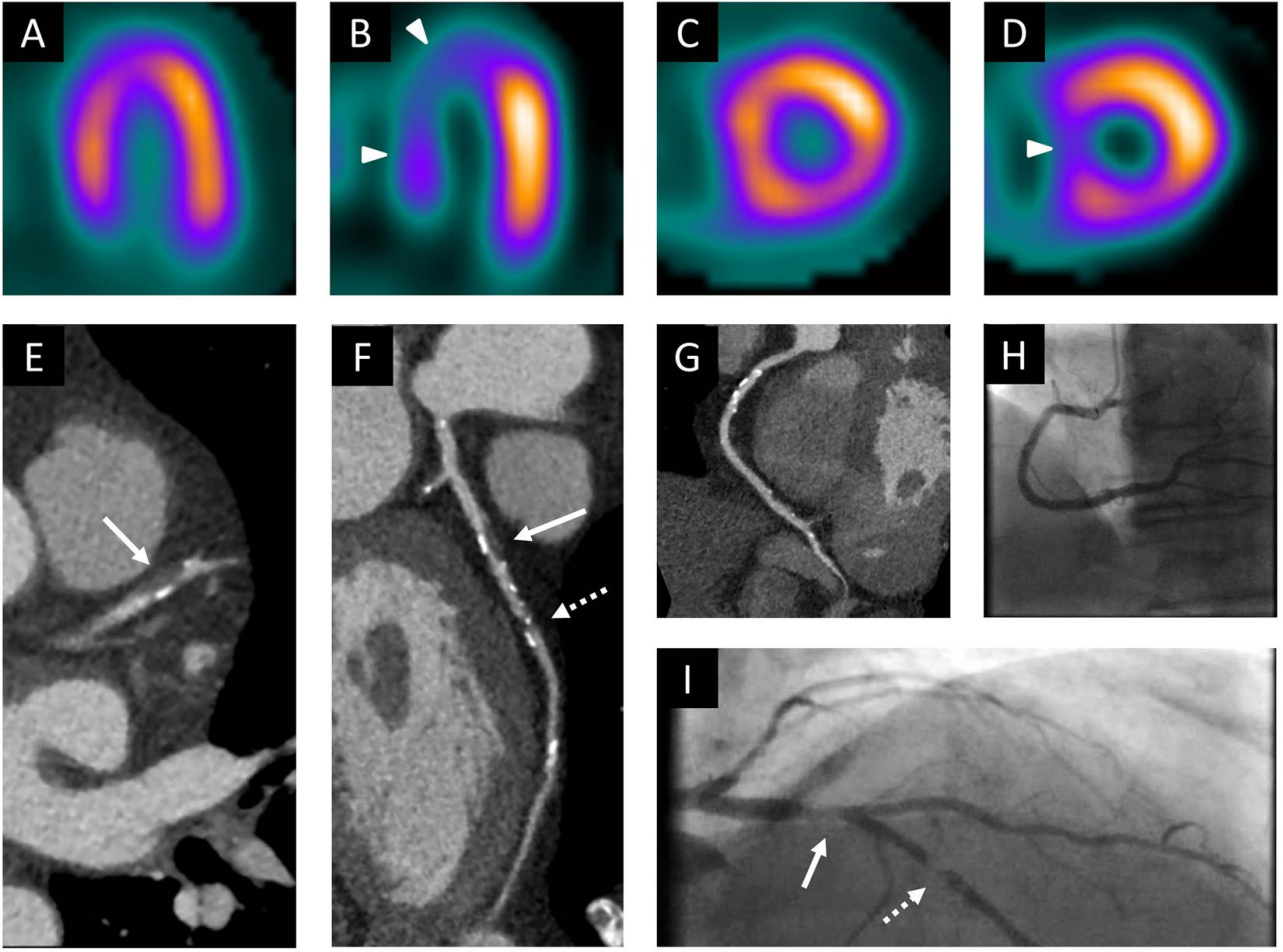
Example of a true positive CCTA examination. This 60 year old male patient underwent stress nuclear myocardial perfusion imaging because of recurrent chest pain. Stress images in the vertical short axis (**B**) and horizontal short axis views (**D**) are suggestive of decreased perfusion to the anterior, septal and apical portions of the left ventricular myocardium (arrowheads) and are largely reversible at rest (**A,C**). The examination was thus interpreted as concerning for ischemia involving the anteroseptal wall and the cardiac apex. At CCTA, transverse section (**E**) and curved multiplanar reformat (**F**) of the left anterior descending artery demonstrate a severe proximal stenosis caused by a predominantly non-calcified plaque (arrow). Another severe stenosis with mixed plaque is noted further distally (dotted arrow). The right coronary artery (**G**) and the circumflex artery show diffuse calcified and non-calcified plaque but no ≥50% stenosis. Absence of critical stenosis in the right coronary artery was confirmed on the catheter angiogram (**H**). The angiogram also confirmed the two critical stenoses in the left anterior descending artery (**I**). Both lesions were successfully treated with angioplasty and stent placement. CCTA = coronary computed tomography angiography.

**Figure 3:**
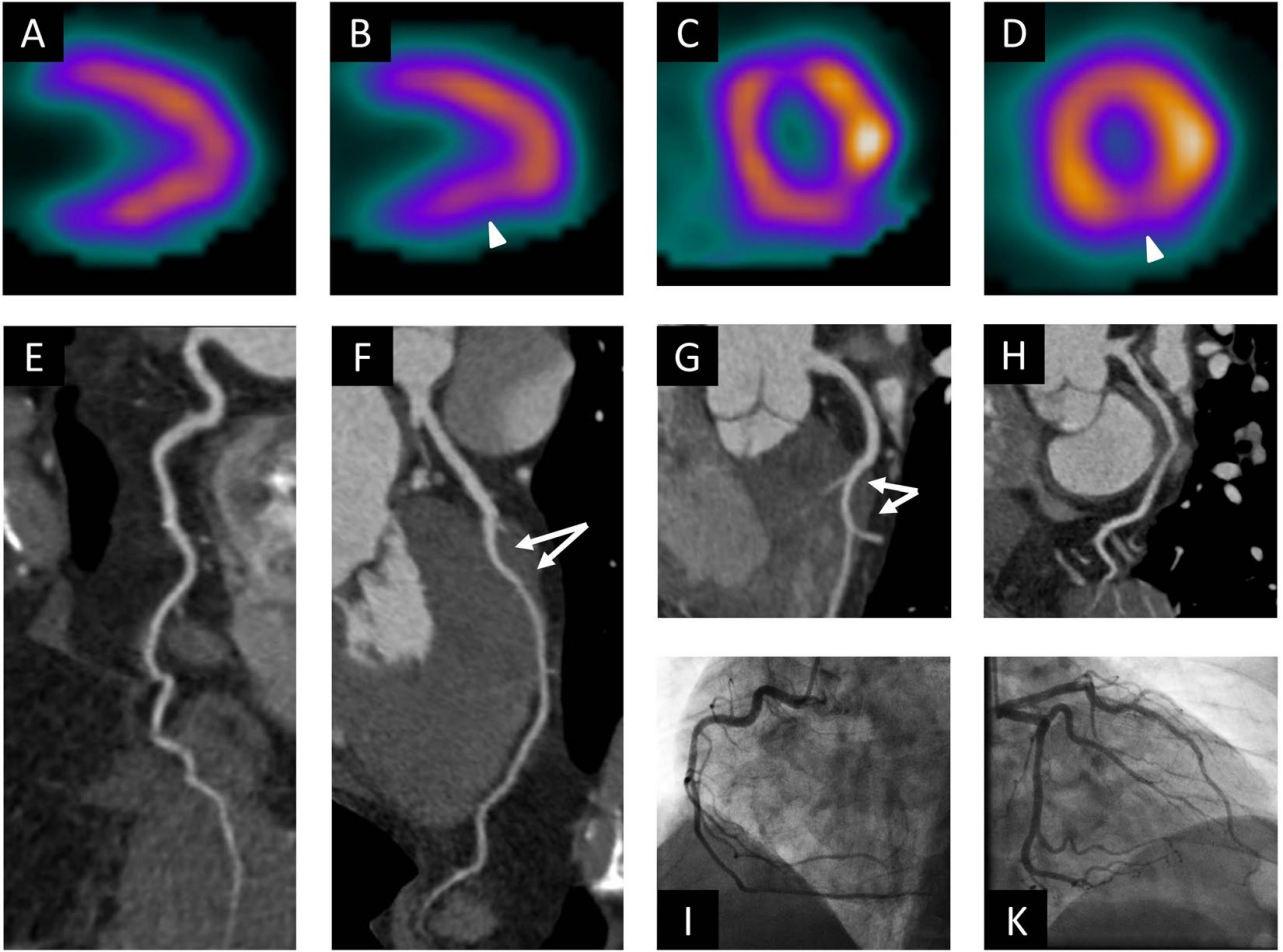
Example of a true negative CCTA examination. This 58 year old male patient underwent stress nuclear myocardial perfusion imaging because of chest pain and dyspnea on exertion. Stress images in the vertical long axis (**B**) and horizontal short axis views (**D**) are suggestive of decreased perfusion to the inferior wall of the left ventricle (arrow heads), which are at significantly reversible at rest without extracardiac activity (**A,C**). The examination was thus interpreted as concerning for inferior wall ischemia. At CCTA, curved multiplanar reformats of the right coronary artery (**E**), left anterior descending artery (**F,G**) and circumflex artery (**H**) demonstrate no evidence of stenosis. Myocardial bridging of the left anterior descending artery with a relatively long intra-myocardial course as a potential cause of the patient’s symptoms is noted (arrows in **F,G**). On catheter angiograms (**I,K**), there is no evidence of coronary artery disease. CCTA = coronary computed tomography angiography.

**Figure 4:**
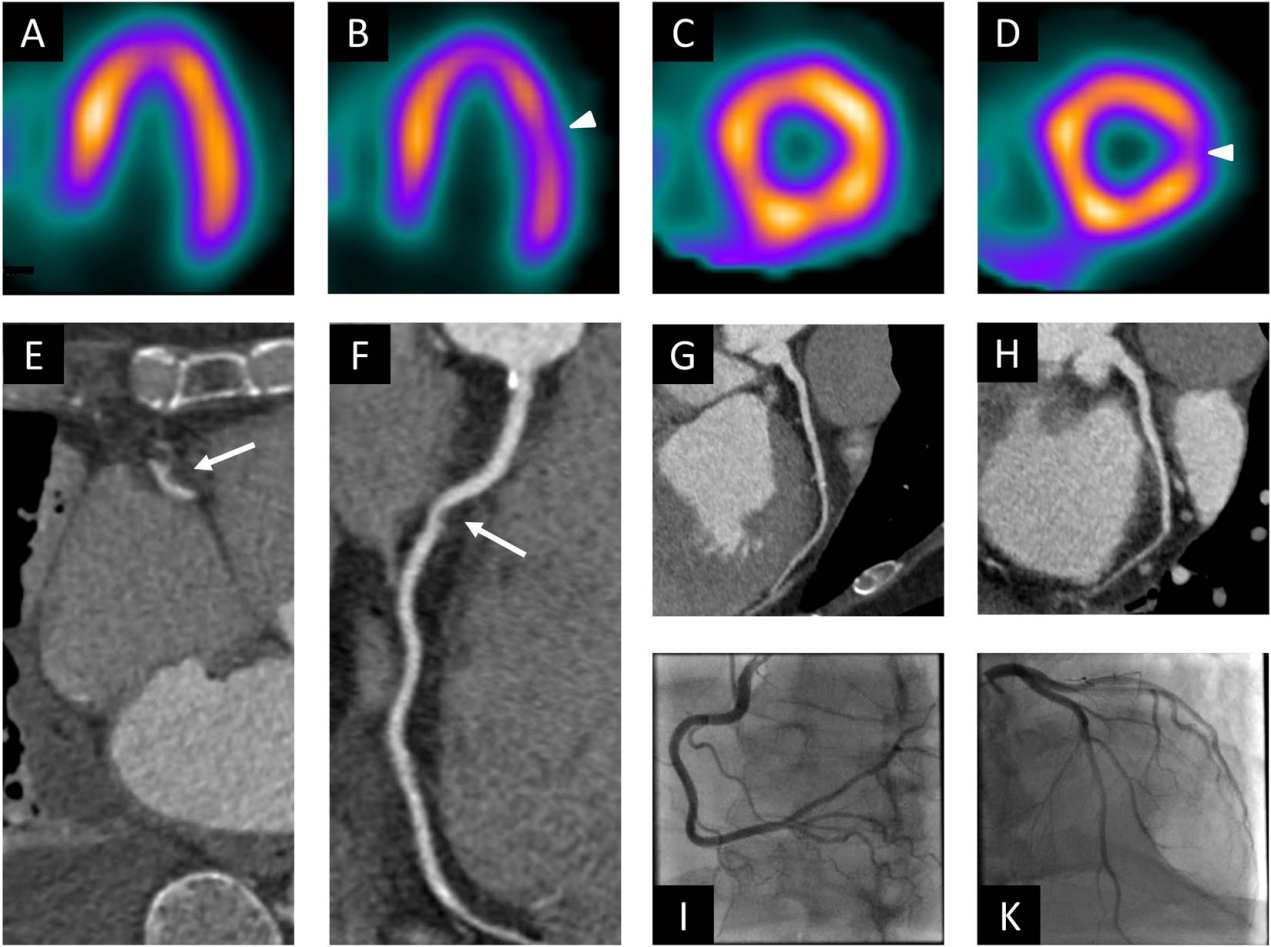
Example of a false positive CCTA examination. This 63 years old male patient underwent stress nuclear myocardial perfusion imaging because of exertional dyspnea. Stress images in the vertical short axis (**B**) and horizontal short axis views (**D**) are suggestive of decreased perfusion to the lateral wall of the left ventricle (arrow heads), which is reversible at rest (**A,C**). The examination was thus interpreted as concerning for lateral wall ischemia. At CCTA, transverse section (**E**) and curved multiplanar reformat (**F**) of the right coronary artery were interpreted as showing 50% stenosis with focal soft plaque (arrow). No luminal narrowing is noted in the left anterior descending (**G**) and circumflex (**H**) arteries. On catheter angiograms (**I,K**), there is no evidence of luminal narrowing in any vessel. The false positive finding on CCTA likely represents an artifact. CCTA = coronary computed tomography angiography.

The flowchart in Fig. 1 illustrates the diagnostic accuracy of CCTA based on the relationship between findings at CCTA and ICA. Images from a representative patient with a true positive finding an MPI, CCTA, and ICA is shown in Fig. 2 for severe stenosis of the left anterior descending coronary artery. Likewise, an example of a true negative correlation between CCTA and ICA after an abnormal MPI is shown in Fig. 3. An example of a false positive CCTA for obstructive CAD not verified with ICA is illustrated in Fig. 4.

## Discussion

The clinical utility of CCTA in the setting of equivocal MPI results has long been recognized, but its role in patients with a higher pretest probability of CAD based on clinical factors and an abnormal MPI has not been well established^3^. Functional and anatomical testing for CAD show only moderate correlation^4–6^ which leads to a relatively low prevalence of obstructive CAD on ICA even after positive stress testing^1,6,7^. This decreases the percentage of ICA patients identified that require coronary angioplasty or surgical therapy. Our prospective study evaluated the diagnostic yield and accuracy of CCTA in higher risk patients independently referred for ICA based on clinical concern for CAD plus an abnormal MPI.

We found almost half of patients (46/100) with an abnormal MPI for suspected CAD demonstrated no potentially obstructive CAD on CCTA. Our data suggests that CCTA has high sensitivity and excellent negative predictive value for clinically significant CAD even in this relatively high-risk cohort of patients. It is possible a significant number of ICA procedures could potentially be safely avoided if CCTA was used as the next diagnostic step in this patient population. Such an approach serially takes advantage of the strengths of each test; i.e., CCTA can effectively rule out anatomic coronary disease whereas functional stress testing may predict risk but cannot fully determine obstructive coronary disease^8^.

Given the clinical importance of identifying obstructive CAD, several studies have evaluated CCTA after abnormal stress test findings in different groups. Many investigations have been limited by a low prevalence of obstructive CAD in the populations studied. For example, the Advanced Cardiovascular Imaging Consortium (ACIC) registry included 3,623 patients with abnormal stress test results but reported >50% stenosis in only 19.7% of patients^7^. Similarly, two retrospective cohort studies of patients with equivocal or abnormal findings on MPI reported a 27–32% prevalence of ≥50% stenosis on CCTA^9,10^. This difference may be due to referral bias, where patients with an abnormal stress test result were preferentially referred for CCTA if a false positive stress test was suspected on clinical grounds^11^. In contrast, our study prospectively enrolled patients with a recent abnormal MPI result and a clinical indication for ICA; thereby avoiding the preferential referral of low-risk patients.

Other previous studies have had inconsistencies regarding the diagnostic accuracy of CCTA compared with ICA in patients with prior stress testing. Again in the ACIC registry, a subgroup of 621 patients who underwent both CCTA and ICA after stress testing had substantially lower values for sensitivity, specificity, PPV and NPV of 93.7%, 37.9%, 70.6% and 79.1% respectively^7,11^. Conversely, one small prospective study (n = 32) in patients who had abnormal MPIs reported 100% sensitivity and 81% specificity for obstructive CAD, which is similar to our results^6^. In part, these discrepancies in accuracy between some of these clinical studies may be attributable to referral biases as an inherent limitation of retrospectively pooled registry data. The lower sensitivity described in the ACIC registry may also be attributed to using >50% stenosis on ICA as the reference marker^7^. For our primary analysis, we used any ≥70% epicoronary stenosis or ≥50% left main stenosis on ICA to define clinically significant CAD, since these thresholds typically prompt consideration for intervention in clinical practice^12^. Of course, it can be argued that any coronary stenosis that leads to a perfusion abnormality is by definition of some hemodynamic significance regardless of the anatomical degree of stenosis. Therefore, absence of obstructive coronary artery stenosis on CCTA and ICA after abnormal nuclear MPI does not necessarily imply that these patients do not have ischemia or a lesser degree of CAD, it does, however, exclude lesions that constitute a contemporary indication for coronary intervention.

The PROMISE (Prospective Multicenter Imaging Study for Evaluation of Chest Pain) trial examined the relative roles of CCTA and stress testing in over 10,000 patients being evaluated for angina symptoms^13^. After 2 years of follow-up the composite endpoint of death, myocardial infarction, angina, hospitalization, or procedural complication occurred in 3.3% and 3.0% of the CCTA and stress testing groups, respectively. Since these results did not reach statistical significance, it remains unclear whether initial stress testing or CCTA as part of a chest pain evaluation yields the best outcome.

The SCOT-HEART study enrolled 4146 patients with stable chest pain to evaluate CCTA in addition to standard care^14^. The CCTA cohort demonstrated an increased frequency and certainty of diagnosing CAD with a secondary endpoint of reduced cardiovascular death in the CCTA group (presumably a reflection of better diagnosis-directed therapy).

Also, similarly to the PROMISE trial, more than two thirds of noninvasive testing performed in patients with stable chest pain showed non-obstructive coronary disease, supporting the role of CCTA in diagnosing patients who may not need coronary intervention^13,15^.

The EVINCI study enrolled 475 patients with stable chest pain to either CCTA or functional stress testing with abnormal results being referred for ICA^16^. Significant CAD was found in 29% of patients as defined by ICA as a >70% stenosis in the major epicoronaries or > 50% in the left main coronary artery. CCTA was found more accurate for detecting significant CAD with a positive predictive value of 83% and negative predictive value of 91%. However, patients received one diagnostic study, not both, and not every patient underwent ICA. In another recent prospective study, the PICTURE trial compared CCTA to MPI in 230 patients with stable chest pain using ICA as a reference standard^17^. CCTA had a superior diagnostic accuracy for detecting >50% and >70% coronary stenosis (for >50%, sensitivity was 92% vs. 54.5% for >70% and specificity 87% vs. 81.5%). However, although all patients underwent CCTA and MPI, only 44 of 230 patients had ICA (i.e., only those with either an abnormal CCTA or MPI), which limits interpretation. Our trial results are compatible with these prior studies but examine results to a higher risk cohort since all patients had positive MPIs and all were evaluated by ICA.

Studies of patients with higher prevalences of CAD are limited. One subgroup of the CORE-64 investigation examined the diagnostic accuracy of MPI versus CCTA in 63 patients with coronary calcium Agatston scores over 400. The comparators for CCTA versus MPI were 0.93 and 0.85 for sensitivity; 0.95 and 0.45 for specificity and 0.88 and 0.63 for negative predictive value respectively for coronary lesions greater than 50% by ICA^18^. In an important single-center study, 340 patients with suspected CAD where ICA was felt clinically indicated were randomized to CCTA or ICA^19^. CCTA reduced the need for ICA from 100% to 14% and increased the diagnostic yield of obstructive CAD on ICA, although not all patients received ICA. Major cardiovascular event rates were similar with the two diagnostic strategies after 3.3 years. CCTA was felt a safe gatekeeper for improving ICA diagnostic yield. Again, our study supports these findings but in a related cohort of higher risk patients whose initial symptoms were anginal or anginal-equivalent determined in the outpatient clinical setting and who were even more selected to have significant CAD since they also had a positive MPI prior to randomization.

Patients with a low pre-test probability of CAD and mild abnormalities on stress testing may not be referred for ICA in contemporary clinical practice but would instead have further non-invasive testing or medical management. Our study design ensured that such cases were not included and that the patient population was thus more representative of patients who indeed undergo ICA in clinical practice in the U.S.A. Put another way, designing optimal CAD testing strategies requires different patient groups to be evaluated. Our study adds a higher risk patient cohort to this evolving paradigm that has the potential to improve patient selection whether the goal is revascularization or risk stratification.

The results of our study should be interpreted in light of certain limitations. We did not perform invasive fractional flow reserve measurements because of lack of uniform availability but instead used anatomic analysis of stenosis severity at ICA as the reference standard in this study. Since patients were recruited after being already referred to ICA by independent clinicians after an abnormal MPI, this served as a control that the degree of ischemia was considered significant. Also, attenuation correction and summed stress scores were not routinely availability. In this sense, this use of SPECT results represents common real world practice. Since the referral for ICA based on abnormal MPI results was not controlled for (since it was the entrance point for inclusion), it is possible different clinical organizations may have differences in their ICA referral rates or local imaging expertise that would affect the generalizability of our results. We could not present a range of values for the presence of non- obstructive disease at ICA as other studies have since our study design focused on quantifying potentially obstructive coronary disease comparatively across the three imaging modalities. Likewise, although determining differences in risk factors for patients with or without abnormal CCTAs would have been useful, this study was not powered adequately to perform such analysis. Since most patients were male (as were the vast majority of patients at the Veterans Affairs Medical Center they were recruited from) a gender bias cannot be excluded. Likewise a bias regarding ethnic origin cannot be excluded since 69% were of Caucasian descent.

One implication of our findings, in concert with other studies, would be the role of beginning with CCTA in symptomatic patients and subsequently adding functional testing if the potential hemodynamic significance of the findings were unclear. Nuclear or other functional stress testing is still the most common diagnostic test performed for symptoms suggestive of coronary disease in the U.S.A. Here we demonstrate potential benefit in a higher risk population within the largest integrated health care system in the U.S.A.

This approach may become even more important as future functional CT technologies are developed. For example, myocardial CT perfusion imaging combined with CCTA has been validated against anatomic and physiologic reference standards^20^ and MPI^21^ and has added predictive value for future cardiovascular events^22,23^.

In conclusion, this prospective study found that almost half of patients clinically referred for ICA after an abnormal nuclear MPI and clinical symptoms demonstrated no potentially obstructive lesions on CCTA. In this setting of patients of higher CAD risk, CCTA had high sensitivity (100%), specificity (84%) and negative predictive value (100%) for obstructive CAD and can thus be considered as a gatekeeper for ICA in this patient population.
